# Restrained eating and self-esteem in premenopausal and postmenopausal women

**DOI:** 10.1186/s40337-014-0023-1

**Published:** 2014-10-14

**Authors:** Suzana Drobnjak, Semra Atsiz, Beate Ditzen, Brunna Tuschen-Caffier, Ulrike Ehlert

**Affiliations:** Department of Psychology, Division of Clinical Psychology & Psychotherapy, University of Zurich, Binzmuehlestrasse 14/26, Zurich, 8050 Switzerland; Department of Psychology, Division of Clinical Psychology & Psychotherapy, Albert-Ludwigs University, Engelbergerstrasse 41, Freiburg, 79106 Germany; Institute of Medical Psychology in the Center for Psychosocial Medicine, University Hospital Heidelberg, Bergheimer Str. 20, Heidelberg, 69115 Germany

**Keywords:** Menopause, Middle age, Restrained eating, Self-esteem, Eating behavior

## Abstract

**Background:**

There has been limited research about disordered eating in middle-aged women, and to date, few data exist about restrained eating behavior in postmenopausal women. Therefore, the aim of this study was to examine eating behavior with a specific focus on menopause as an associated factor in restrained eating. Beyond this, we were interested in how postmenopausal status and self-esteem would interact to determine eating patterns in women in middle age.

**Methods:**

We conducted an online survey in women aged between 40 and 66. Eating behavior was assessed with the Eating Disorder Examination-Questionnaire (EDE-Q) in premenopausal (N = 318) and postmenopausal women (N = 250). All participants rated their self-esteem using the Rosenberg Self-Esteem Scale (RSE) and reported their weight, height, waist circumference, and hip circumference.

**Results:**

15.7% of all participants showed clinically meaningful scores on restrained eating. Postmenopausal women showed significantly higher scores on the EDE-Q subscale of restrained eating as compared to premenopausal women, but when controlling for body mass index, however, this finding was no longer significant. Further exploratory analyses suggest that particularly low or high self-esteem levels are associated with restrained eating. Self-esteem might serve as a mediator between menopausal status and restrained eating, however results of these additional analyses were inconsistent.

**Conclusions:**

Restrained eating may appear in middle-aged women. Particularly in postmenopausal women, restrained eating might be associated with lower and higher self-esteem.

## Background

An increasing number of middle-aged women from highly industrialized countries are practicing disordered eating behaviors [1]. Eating disorders often show a chronic course, functional impairment, and seem to increase the risk of several health problems such as future obesity, depressive disorders, suicide attempts, anxiety disorders, and substance abuse [2]. Besides clinically diagnosed eating disorders such as anorexia nervosa and bulimia nervosa [3], disordered eating includes a wide range of eating-related problems [4]. Such behavior occurs relatively frequently but does not meet full criteria for the diagnosis of an eating disorder [4,5]. In general, disordered eating is a well-researched phenomenon, which is mainly assessed in adolescent and young adult women [3]. So far, few studies have investigated disordered eating in women between the ages of 40 and around 65 years [6], although health professionals have recently begun to focus their attention on disordered eating among this age group [7]. Admissions of middle-aged women to eating disorder inpatient treatment has increased over the last ten years [8,9].

Although in several studies, restrained eating has been identified as a main risk factor for young women to develop an eating disorder [10], as yet, specific trajectories of eating disorders in middle-aged women remain unknown. In general, restrained eating is defined as an intentional restriction of energy intake with the aim of losing or controlling weight [11]. Despite this definition, several studies indicate that restrained eating predicts weight gain and might be a risk factor for future onset of obesity [12,13].

Several studies found biological (e.g. body mass index), psychological (e.g. self-esteem), and sociocultural influences (e.g. weight-related teasing) [1] to predict disordered eating patterns – and these seem to be independent of age. However, a specific factor associated with eating behavior in middle-aged women might be menopause status. Menopause is defined as the permanent cessation of the primary functions of the human ovaries [14]. Menopause can be determined retrospectively, once 12 months have elapsed with no menstrual cycle, and this time frame distinguishes menopause from other related terms such as premenopause, perimenopause and postmenopause [14]. Premenopause describes a period of time with regular menstrual cycles. Perimenopause is a period of time when menstrual cycles begin to become irregular. Finally, postmenopause starts after the final menstrual period. After 12 months of amenorrhea, it can be determined that a woman has entered postmenopause [14].

Menopausal transition is a complex psychophysiological process [14]. During the menopausal transition, women could gain an average of 2 to 2.5 kg in weight over 3 years, which is often regarded as the major contributing factor to midlife body weight gain in women [15]. Besides this, it has been shown that a shift to an abdominal fat redistribution occurs [16]. Since slimness is an important criterion of female attractiveness in many societies [17,18], it can be assumed that middle-aged women may experience a loss of attractiveness due to the age-related naturally occurring weight gain [19]. However, the data regarding body image in this age group are contradictory. Although researchers found that the perception of attractiveness is not influenced by age or menopausal status [19], postmenopausal women reported less positive attitudes about their appearance than premenopausal women [20].

Overall, body weight and female self-experienced attractiveness are closely connected, as are self-rated attractiveness and self-esteem [21]. Additionally, as supported by meta-analyses, self-esteem seems to be associated with disordered eating [22]. Lower self-esteem, which means a reduced sense of contentment, self-acceptance, and a reduced appraisal of one’s own worth, predicts higher rates of eating disorders in young women [22-25] and body dissatisfaction or symptoms of bulimia nervosa in middle-aged women [7,26,27].

Our study was designed with two aims: first, to examine the association between menopausal status and disordered eating, with a particular focus on restrained eating in middle-aged women. Our hypothesis was that postmenopausal women will show increased scores in disordered eating than premenopausal women. Our second aim was to examine the role of self-esteem in disordered eating in this age group. We hypothesized that low self-esteem will be positively associated with disordered eating in postmenopausal women.

## Methods

### Data collection and participants

Potential participants were contacted by email using mailing lists and websites from women’s organizations (e.g. www.frauenlandsgemeinde.ch) in Switzerland. The information of the advertisement in the internet in order to inform and recruit the women contained a brief explanation about the study: We introduced the background of the study with the fact that little has been known about eating behavior and related factors in middle-aged women. After this short introduction we informed potential study participants that the online questionnaire would contain questions about demographics, their eating behavior, psychological variables, and menstrual cycle status. Email recipients were also informed that the online survey would take about 15 minutes. The inclusion criteria was age over 40 and female gender. At the end of our information letter, we referred to the link, which had to be opened in order to get to the questionnaire. The survey was programmed using the software *Unipark* (http://www.unipark.info/1-0-online-befragungssoftware-fuer-studenten-und-universitaeten-unipark-home.htm). Overall, we sent about 5’000 Email invitations and 1101 potential participants started to fill out our questionnaire. The drop-out rate (participants who did not fill out the questionnaire completely) in our study was 40.2%.

Participation in the study was voluntary and all women gave their informed consent. Only participants who agreed with the informed consent were able to fill out the questionnaire. Upon completion of the questionnaire, the participants were thanked for their filling in the questionnaire and informed that their anonymity would be guaranteed. Participants who were interested in a follow-up study or who wished to receive further information on the study results could leave their e-mail addresses. Additionally, contact information was provided for the unlikely event that responding to stress or symptom-related questions caused acute psychological problems. This study was conducted in accordance with rules and regulations of the appropriate ethics committee (The Faculty of Arts and Humanities of the University of Zurich, Switzerland).

**Figure 1:**
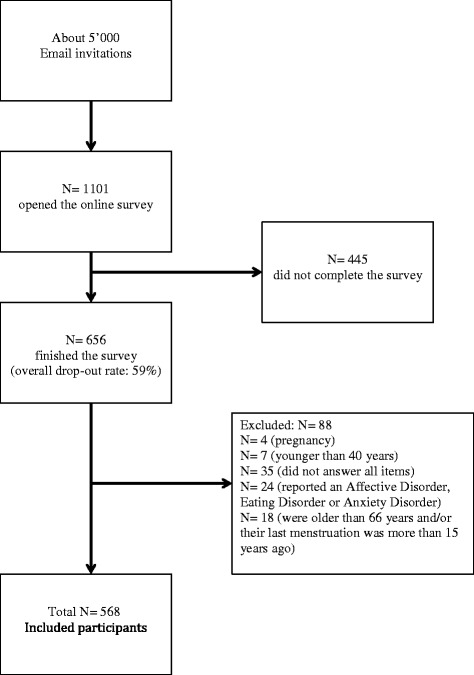
**Process of data selection.**

We excluded participants with the following criteria: age under 40 or over 66, pregnancy, self-reported Affective Disorder, Eating Disorder or Anxiety Disorder, last menstruation more than 15 years ago and participants who did not complete all questions. After the completion of the study, menopausal status was defined as follows: premenopausal women (N = 318) reported to have a regular or irregular menstrual cycle in the past 12 months, which means that participants with a possible perimenopausal status were in the group premenopausal women included, too. Postmenopausal women (N = 250) had not had a menstrual cycle during the past 12 months. The women were asked to provide the date (day, month, year) of their last menstruation. The detailed process of recruitment and the selection of the two groups from a large sample are shown in Figure 1.

## Assessment measures

The online survey consisted of 58 items. The first section of questions comprised items addressing socio-demographic, general health-related, and menstrual cycle-related variables. The second section of the survey consisted of questions about eating behavior and self-esteem, presented in a randomized order.

### Body Mass Index and Waist-to-Hip Ratio

Participants were asked to indicate their weight and height, and BMI was calculated as weight divided by the square of height in cm. Besides this, participants were asked to measure their waist (at the midpoint between the lower margin of the last palpable rib and the top of the iliac crest) and hip circumference (widest portion of the buttocks) [28]. Waist-to-hip ratio (WHR) was calculated by dividing the waist by the hip circumference [28]. In addition, we illustrated the definition of the WHR measurement with a picture. WHO norms [28] define obesity for females as a WHR above 0.85, or a BMI above 30). BMI and WHR were used as control variables in this study.

### Eating behavior

Eating behavior was assessed with the German version of the Eating Disorder Examination-Questionnaire (EDE-Q) [29,30]. The EDE-Q is a self-report instrument with 36 items covering the previous 28 days. It contains four subscales measuring restrained eating, shape concern, weight concern, and eating concern, to be summarized with a global score. The items can be answered on a 7-point scale, with higher scores reflecting greater eating-related pathology. Frequencies of disordered eating behaviors including binge eating and various compensatory behaviors are also assessed. Based on this questionnaire, “extreme dietary restraint” was defined with reference to the EDE-Q “food avoidance” item, namely “going without food for a period of eight or more waking hours on average three or more times per week in order to influence weight or shape” [31,32]. For the EDE-Q, norms for anorexia nervosa, bulimia nervosa, and eating disorders not otherwise specified are available for young women [29,30,33]. The scores of the subscale “restrained eating” range from 0.1 to 2.6 among women without diagnosed eating disorders, while means of restrained eating between 2.7 and 6 are found in women with eating disorders. Women with clinically meaningful scores show values ranging between 3.7 and 6.0 for “shape concern”, between 3.09 and 6.0 for “weight concern”, and between 1.85 and 6 for “eating concern” [30].

Additional six items measure diagnostically relevant information, e.g. symptoms of binge eating. In our study, we analyzed the item 13 “Over the past 28 days, how many times have you eaten what other people would regard as an unusually large amount of food (given the circumstances)?” and item 14 “On how many of these times did you have a sense of having lost control over your eating (at the time you were eating)?”. These key behavioral items were dichotomized for any occurrence (≥1 episode versus 0 episodes over the past 28 days) and regular occurrence (≥4 or < 4 episodes in the last 28 days) [31].

In this study, the Cronbach’s alpha for each subscale was acceptable (restrained eating = .78; shape concern = .89; weight concern = .77; eating concern = .82).

### Self-esteem

Self-esteem was assessed with the German version [34] of the self-esteem scale (RSE) [35], which is a widely used self-esteem inventory in social science research. The scale contains 10 items to be scored from 1 = “totally disagree” to 4 = “totally agree”. Negatively coded items were recoded in order to calculate the sum score. In the present study, the Cronbach’s alpha for SES was .74, which is acceptable.

### Data analysis

Following overall descriptive data analysis and the analysis of frequencies and intensities of disordered eating behavior, and the key behavioral factors for binge eating, premenopausal women and postmenopausal women were compared with regard to eating behavior, such as restrained eating, shape concern, eating concern and weight concern, using multivariate ANOVAs. Furthermore, BMI, age and WHR were included as covariates, and univariate ANOVAs with menopause status as factor and restrained eating or self-esteem as dependent variables were conducted. An additional univariate ANOVA was performed with menopause status as factor and “extreme dietary restraint” as dependent variable including the control variables BMI, age and WHR.

## Results

### Participants’ characteristics

Table 1 provides descriptive data of the total study sample (N = 568 women, aged from 40 to 66 years).

**Table 1:** **Characteristics of study population**

**Characteristics**	**Premenopausal (N = 318)**	**Postmenopausal (N = 250)**	**Group difference t-test/Chi-square**
Age	M = 46.40, SD = 3.96	M = 54.98, SD = 5.19	T = −22.499, p = .000
Education			
Any type of college and higher	N = 228 (71.7%)	N = 139 (49.6%)	χ^2^ = 6.698, p = .153
Marital status			
Married/in a relationship	N = 200 (62.9%)	N = 190 (76.0%)	χ^2^ = 0.536, p = .464
Single/separated/divorced	N = 118 (37.1%)	N = 60 (24.0%)	χ^2^ = 1.002, p = .317
Employment	76-100%	N = 152 (47.8%)	N = 95 (38.0%)
unemployed/up to 75%	N = 166 (52.2%)	N = 155 (62.0%)	χ^2^ = 3.003, p = .391
Children	M = 1.4, SD = .49	M = 1.26, SD = .44	T = −1.144, p = .253
Months after beginning of the last menstruation	M = 1.00, SD = 1.79	M = 97.81, SD = 68.36	T = 24.497, p = .000
BMI	M = 23.34, SD = 3.86	M = 23.74, SD = 4.53	T = −1.133, p = .258
WHR	M = 0.812, SD = 0.07	M = 0.828, SD = 0.09	T = −1.758, p = .080
EDE-Q__restrained_	M = 1.23, SD = 1.32	M = 1.45, SD = 1.35	T = −1.983, p = .048
EDE-Q__eating_	M = 0.46, SD = 0.87	M = 0.48, SD = 0.82	T = − 0.295, p = .768
EDE-Q__weight_	M = 1.28, SD = 1.29	M = 1.29, SD = 1.27	T = − 0.080, p = .936
EDE-Q__shape_	M = 1.63, SD = 1.45	M = 1.67, SD = 1.47	T = − 0.305, p = .761
RES	M = 39.77, SD = 4.64	M = 38.86, SD = 4.82	T = 2.288, p = .023

*Note:* EDE-Q _restrained_ = subscale restrained eating of the EDE-Q; EDE-Q _eating_ = subscale eating concern of the EDE-Q; EDE-Q _weight_ = subscale weight concern of EDE-Q; EDE-Q _shape_ = subscale shape concern of the EDE-Q; BMI = body mass index; RSE = Rosenberg Self-Esteem Scale.

### Frequencies and intensities of disordered eating behavior

80.8% (N = 459) of all participants show a score higher than 0 on restrained eating. 15.7% (N = 116) of the participants show clinically relevant scores on restrained eating. 10.7% (N = 61) participants reported “Extreme dietary restraint”, defined as going without food for a period of eight or more waking hours on average three or more times per week. Of these 61 participants, one participant reported a BMI < 18.5, 42 a BMI between 18.5 and 25, and 18 women reported a BMI over 25.

**Figure 2:**
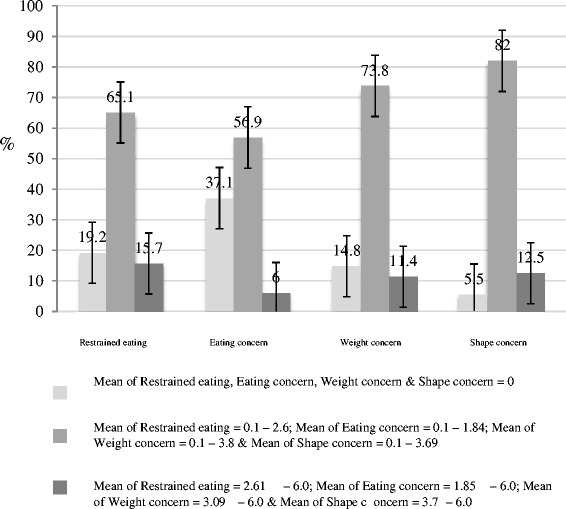
**Manifestations of disordered eating behavior.**

Comparable scores were found in the subscales eating concern, weight concern and shape concern, with 56.9% to 82% of all participants reporting low to moderate manifestations of disordered eating. 6% to 12.5% reported clinically meaningful scores on these subscales. Figure 2 displays the distributions of eating patterns.

In addition, results of the key behavioral features showed the following results: 69.7% (N = 396) showed no occurrence of eating large amounts of food (EDE-Q item 13), whereas 30.3% (N = 172) scored higher than 1. 86.7% (N = 493) reported no regular occurrence of this behavior whereas 13.3% (N = 75) showed scores higher than 4. 77.5% (N = 440) showed no occurrence of lost control during eating (EDE-Q item 14), whereas 22.5% (N = 128) scored higher than 1. 89.2% (N = 507) reported no regular occurrence of this behavior whereas 10.8% (N = 61) showed a score over 4.

### Correlations between eating behavior and associated variables

Most correlations were significant at the .001 level, except for WHR and menstrual status, where the associations with the other variables were mostly insignificant (see Table 2).

**Table 2:** **Correlations between all variables in the study**

		**1**	**2**	**3**	**4**	**5**	**6**	**7**	**M**	**SD**	**Range**
1	m.status	-							-.087	.997	−1, +1
2	RSE	-.096^a^	-						39.351	4.717	17-50
3	EDE-Q _restraint_	.088^a^	-.213						1.340	1.345	0-6
4	EDE-Q _eating_	.005^b^	-.396	.555					. 467	.843	0-6
5	EDE-Q _weight_	.000^b^	-.333	.642	.716	-.333			1.281	1.280	0-6
6	EDE-Q _shape_	.012^b^	-.322	.638	.701	.910			1.651	1.451	0-6
7	BMI	.039^b^	-.043^b^	.200	.267	.456	.427	-	23.495	4.144	14-46
8	WHR	.096^b^	-.027^b^	.032^b^	.063^b^	.142^a^	.186	.283	.8182	0.081	.45-1.27

*Note:* Unless otherwise noted, all coefficients were significant at the p = .001 level; ^a^: significant at p < .05; ^b^: non-significant; m.status = menopausal status; RSE = Rosenberg Self-Esteem Scale; EDE-Q _restraint_ = subscale restrained eating of the EDE-Q; EDE-Q _eating_ = subscale eating concern of the EDE-Q; EDE-Q _weight_ = subscale weight concern of EDE-Q; EDE-Q _shape_ = subscale shape concern of the EDE-Q; BMI = body mass index; WHR = Waist-to-Hip Ratio.

### Group differences

The overall multivariate analyses (Hoteling-Spur) with menopause status as factor and the subscales of EDE-Q and RSE as dependent variables revealed a main effect of menopause status change [F(5, 562) = 2.584, p = .025, partial η^2^ = .022]. Results obtained by multivariate ANOVAs indicate that restrained eating in postmenopausal women is increased compared to premenopausal women (*F*[1, 566] = 3.931, *p = 0.048,* partial η^2^ = .007), and self-esteem is decreased in postmenopausal women (*F*[1, 566] = 5.234, *p = 0.023,* partial η^2^ = .009). No significant differences in other eating behavior variables, such as eating concern (*F*[1, 566] = 0.087, *p = 0.768,* partial η^2^ = .000), weight concern (*F*[1, 566] = 0.006, *p = 0.936,* partial η^2^ = .000), and shape concern (*F*[1, 566] = 0.093, *p = 0.761,* partial η^2^ = .000), were observed between pre- and postmenopausal women (see Table 1). No covariates were included in this multivariate analysis.

Results obtained by univariate ANOVAs with menopause status as factor and BMI as dependent variable indicate that Body mass index (BMI) did not differ between the groups (*F*[1, 566] = 1.284, *p = 0.258,* partial η^2^ = .002). Additionally, WHR did not differ between the groups (*F*[1, 330] = 3.091, *p = 0.080,* partial η^2^ = .009).

Results obtained by univariate ANOVAs with menopause status as factor and restrained eating as dependent variable show that when controlling for BMI, WHR and age, BMI was a significant covariate (*F*[1, 327] = 18.038, *p = 0.000,* partial η^2^ = .052)*.*

The results with the subgroup of women who reported “Extreme dietary restraint” showed a different pattern of findings: The univariate ANOVAs with menopause status as factor and restrained eating as dependent variable show that when controlling for BMI, WHR and age, menopause status (*F*[1,24] = 4.615, *p = 0.042,* partial η^2^ = .161*)* and WHR (*F*[1,24] = 5.406, *p = 0.029,* partial η^2^ = .184*)* were significant predictors of restrained eating.

Results obtained by univariate ANOVAs with menopause status as factor and self-esteem as dependent variable show that when controlling for BMI, WHR and age, menopause status (*F*[1, 327] = 5.789, *p = 0.017,* partial η^2^ = .017) is a significant predictor of self-esteem.

### Associations between self-esteem and restrained eating

#### U-shaped association between self-esteem and restrained eating

**Figure 3:**
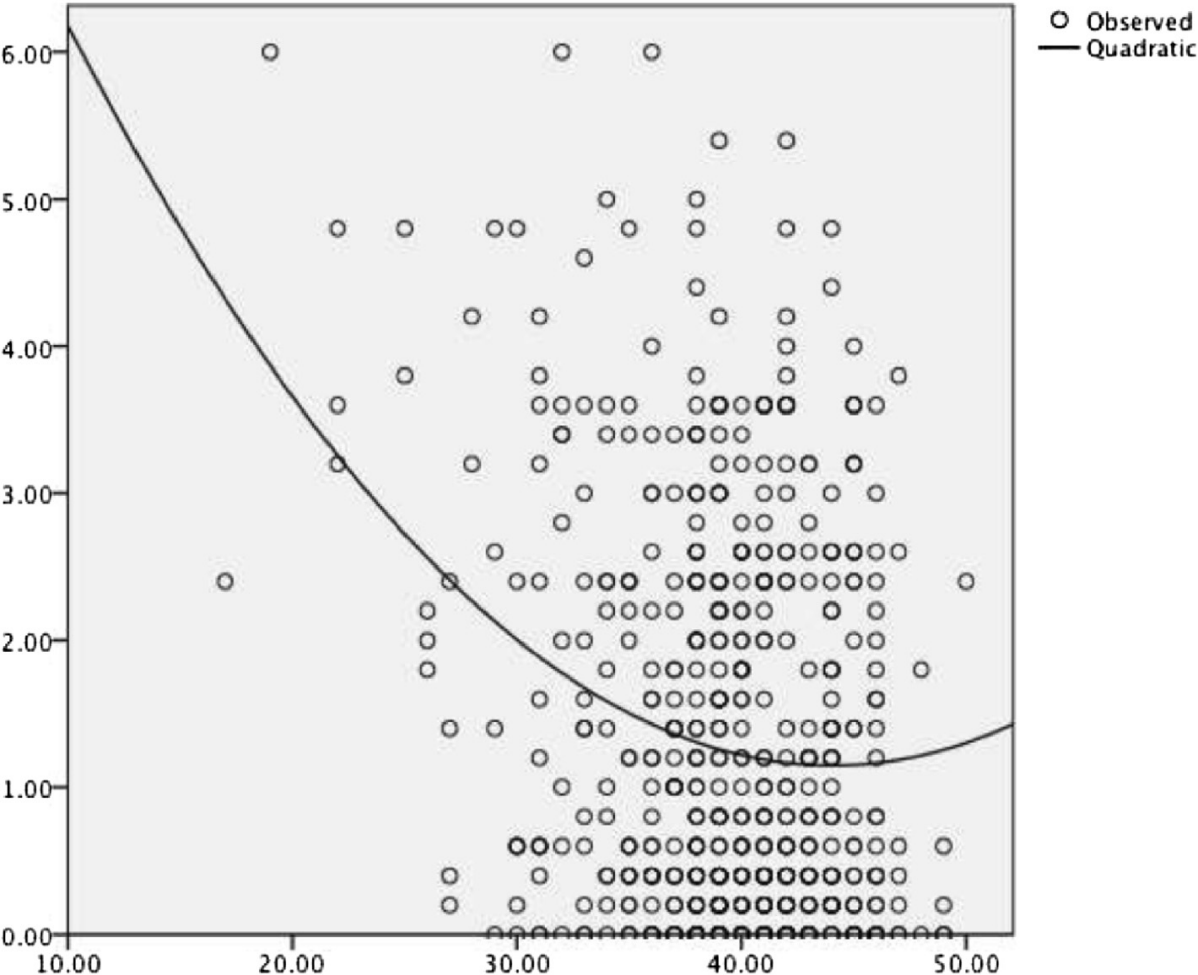
**U-shaped relationship between self-esteem and restrained eating.**

Results from the multiple regression analysis with self-esteem and self-esteem squared as independent variables and restrained eating as dependent variable are shown in Table 3 and suggest a U-shaped relationship (see Figure 3). Overall, self-esteem explained 6.1% of the variance in restrained eating.

**Table 3:** **Multiple regression analysis with restrained eating as dependent variable**

	**Unstandardized coefficients,** ***B***	**SE**	**Standardized coefficients,** ***β***	**Level of significance,** ***p***
Constant	9.566	1.946		.000
RSE	-.382	105	−1.340	.000
RSE^2^	.004	.001	1.134	.002

*Note:* RSE = Rosenberg Self-Esteem Scale.

A mediation analysis with menopause status as an independent, restrained eating as a dependent variable and self-esteem as potential mediator based on the Baron & Kenny [36] and Sobel method [37] suggested that self-esteem mediated the relationship between menopause status and restrained eating. However, the bootstrapping approach by Preacher and Hayes ([38], and as available as an SPSS-syntax on their homepage, http://www.afhayes.com/spss-sas-and-mplus-macros-and-code.html) did not provide significant results. We, thus, do not further interpret the results from the aforementioned mediation analyses.

## Discussion

This study focused on the role of menopausal status and self-esteem in terms of their relationship with eating behavior in normal-weight middle-aged women. Overall, the results suggest that restrained eating is a relavitely frequent behavior among middle-aged women: 15.7% of all participants report restrained eating scores in a clinically meaningful range. In addition, 10.8% - 13.3% of all participants showed a regularly occurrence of binge eating symptoms. Compared to premenopausal women, postmenopausal women report a decreased self-esteem and higher levels of restrained eating, whereas this effect disappears when BMI and age are controlled for. However, even when controlling for BMI, WHR and age, both menopause status and WHR seem to be in a relationship with extreme dietary restraint. Self-esteem was associated with restrained eating, showing a U-shaped curve. Based on these data, it might be followed that self-esteem mediates the relationship between menopausal status and restrained eating, however the results of the additional mediation analyses were inconsistent.

The assessed mean scores of the subscales of the EDE-Q indicate that middle-aged women’s eating concerns are comparable with those of young adult women [31]. However, to date, no normative data for this older age group are available. In the present study, a subsample of 6–15.7% women showed subscale scores in a clinically meaningful range, thus suggesting that negative body image and eating disorders may appear in older women [39,40].

Even tough a recent study examined overweight middle-aged participants and showed increased restrained eating in postmenopausal women, our finding that normal-weight postmenopausal women show increased restrained eating when not controlling for BMI could be seen in a similar line [41]; we have also found an associtation between menopausal status and restrained eating.

The authors described that postmenopausal women (M = 51.7 years) reported higher levels of dietary restraint than premenopausal women (M = 35.7 years). However, in that study, postmenopausal women still had a significantly higher BMI than premenopausal women. To the best of our knowledge, this is the first study in this field, examining participants with normal weight. Although restrained eating and BMI are modestly positively correlated, it is highly surprising that restrained eating differs between the average normal-weighted pre- and postmenopausal women. This finding might have interesting implications for further studies.

Furthermore, menopause-related changes in body fat distribution or increasing BMI in middle-aged women are seen as having a negative impact on appearance [42-44,25]. Beyond this, the physiological changes after menopause could be associated with increased feelings of body dissatisfaction and lower psychological well-being than in the years before menopause [45,46]. When internalized ideals of appearance no longer seem achievable [45], this may affect body image and eating behaviors. This effect seems to be even greater the more physical appearance is seen as an integral part of the self-concept [46].

It is difficult to distinguish the association between menopause and restrained eating or BMI from age or age-related factors, such as sedentary lifestyle, slowed metabolism, or less sleep. All of these factors have independently been associated with weight gain [47]. Indeed, our data suggest that postmenopausal women showed higher restrained eating and, when controlling for BMI, WHR and age, only BMI seems to be in a modest positive relationship with restrained eating in middle-aged women. Previous research suggests positive [48], negative [49], or no relationships between BMI and restrained eating. However, in normal-weight groups, restrained eating and BMI seem to be positively associated [48,50,51]. It might therefore follow that in normal-weight populations, restrained eating may serve as a marker for the tendency to overeat – which could then call for constant counter-regulation.

In younger women, several studies have shown that decreased self-esteem is a risk factor for developing disordered eating behavior [22]. However, results regarding this relationship in middle-aged women have been inconsistent, with some suggesting a positive and some a negative relationship [26,52]. The U-shaped relationship found in the present study now suggests that low as well as high self-esteem might be associated with restrained eating, although the findings are more robust for the former. This finding seems to integrate the previous and seemingly contradictory results. Many women seem to link their self-esteem to their appearance. The realization that one is no longer able to fulfill societal ideals of appearance was found to have a negative impact on self-esteem [46]. Given that restrained eating is intended to maintain or decrease a particular weight [22], and weight loss was found to improve self-esteem [53], increased restrained eating could be regarded as a measure to raise self-esteem [54].

However, the results should also be interpreted in light of the study’s strengths and limitations. Online-based data collection allows access to a broad sample of participants as well as the assessment of data in a short period of time [55]. Nevertheless, this is a cross-sectional study and the results should be interpreted with caution because our results show only associations without causal meanings; only longitudinal study might provide more conclusive answers to the question of whether menopausal status and self-esteem might cause disordered eating in middle-aged women. Furthermore, potential problems with online recruitment may occur. Online studies are based on self-reported data, which leads to the inability to verify the inclusion and exclusion criteria. In addition, individuals with high BMI or circumferences might not want to share this information, even if it is anonymous. For example, even tough we tried to provide a clear definition of the WHR measurement, it might be subjective what the “widest portion of the buttocks” is, and therefore cause biased results. Overall, the recruited participants might be a biased selective population. It could also explain why the BMI is in a normal weight range. In addition, mixing potential perimenopausal and postmenopausal women may have biased the results – a limitation which is due to our online assessment lacking an endocrine validation of menopausal status. We acknowledge that future and methodological more rigorous studies in this field might use endocrine assessments. However, with this initial investigation in this relatively large sample, endocrine analyses would not have been feasible.

Moreover, participants in this online study may have reported specific eating behaviors as a consequence of their educational and professional background.

## Conclusions

In conclusion, this study focused on menopausal status, self-esteem and eating patterns in middle-aged women, and in so doing, might shed light on this relatively understudied topic. It shows that not only women with diagnosed eating disorders may show eating-related changes, but also non-diagnosed, healthy, middle-aged and normal-weighted women may have increased scores, especially in restrained eating. It remains to be analyzed whether it is specifically menopausal status or other age-related factors, which influence self-esteem in middle-aged women. As described in a recent study, it can be hypothesized that menopausal transition could represent a window of vulnerability to eating-related changes [56]. In Western societies, menopausal transition is often perceived as a time of emotional and physical health impairment [43]. Independently, disordered eating is influenced by a multitude of bio-psycho-social factors [24,57]. Thus, while not every woman is necessarily predisposed to developing restrained eating patterns during her menopausal transition, her experienced self-esteem might influence this relationship. According to recent data, over 70% of women aged over 50 years report dissatisfaction with their current weight and shape, being compared to when they have younger [58], likely, being vulnerable to develop disordered eating. This suggests that women in midlife might be particularly vulnerable to developing disordered eating behavior. Further studies could help identify specific risk groups for disordered eating in this age group, in order to develop prevention and treatment options for the most vulnerable. With increased ageing in our societies and stable youth-related norms of attractiveness, the demand for such interventions might well increase over the coming years.
